# Expressed HNSCC variants by HPV-status in a well-characterized Michigan cohort

**DOI:** 10.1038/s41598-018-29599-w

**Published:** 2018-07-30

**Authors:** Tingting Qin, Yanxiao Zhang, Katie R. Zarins, Tamara R. Jones, Shama Virani, Lisa A. Peterson, Jonathan B. McHugh, Douglas Chepeha, Gregory T. Wolf, Laura S. Rozek, Maureen A. Sartor

**Affiliations:** 1 Department of Computational Medicine and Bioinformatics, University of Michigan Medical School, Ann Arbor, Michigan USA; 2 Department of Environmental Health Sciences, University of Michigan School of Public Health, Ann Arbor, Michigan USA; 3 Department of Otolaryngology-Head and Neck Surgery, University of Michigan Medical School, Ann Arbor, Michigan USA; 4 Department of Pathology, University of Michigan Medical School, Ann Arbor, Michigan USA; 5 Present Address: Ludwig Institute for Cancer Research, 9500 Gilman Drive, La Jolla, CA 92093 USA; 6 Present Address: Department of Otolaryngology, University of Toronto, Toronto, Ontario, Canada

## Abstract

While whole-exome DNA sequencing is the most common technology to study genetic variants in tumors in known exonic regions, RNA-seq is cheaper, covers most of the same exonic regions, and is often more readily available. In this study, we show the utility of mRNA-seq-based variant analysis combined with targeted gene sequencing performed on both tumor and matched blood as an alternative when exome data is unavailable. We use the approach to study expressed variant profiles in the well-characterized University of Michigan (UM) head and neck squamous carcinoma (HNSCC) cohort (n = 36). We found that 441 out of 455 (~97%) identified cancer genes with an expressed variant in the UM cohort also harbor a somatic mutation in TCGA. Fourteen (39%) patients had a germline variant in a cancer-related Fanconi Anemia (FA) pathway gene. HPV-positive patients had more nonsynonymous, rare, and damaging (NRD) variants in those genes than HPV-negative patients. Moreover, the known mutational signatures for DNA mismatch repair and APOBEC activation were attributive to the UM expressed NRD variants, and the APOBEC signature contribution differed by HPV status. Our results provide additional support to certain TCGA findings and suggest an association of expressed variants in FA/DNA repair pathways with HPV-associated HNSCC tumorigenesis. These results will benefit future studies on this and other cohorts by providing the genetic variants of key cancer-related genes.

## Introduction

Whole-genome sequencing (WGS) and whole-exome sequencing (WES) are the optimal approaches to elucidate somatic mutations that alter protein function or abundance in carcinogenesis^1,2^. However, compared to genome sequencing, RNA-seq is more popular due to its lower cost and its ability to address a range of hypotheses, including gene expression quantification, detection of alternative splicing, allele-specific expression, viral integration, gene fusion^3–7^ and RNA editing^8–10^. Therefore, a large number of samples have available RNA-seq data but not WGS or WES data, and calling genomic variants from these already available data can improve the genomic information for these existing samples. Although calling and interpreting variants from RNA-seq data remains challenging due to the intrinsic complexity in the transcriptome, advances in bioinformatics pipelines for RNA-seq variant calling, such as the GATK best practice pipeline tuned for RNA-seq variant detection, provides an alternative way to study genetic variation (https://software.broadinstitute.org/gatk//guide/article?id=3891). RNA-seq also has the disadvantage that nonsense mutations, deletions, and other mutations that destabilize the transcript will not be detected. However, RNA-seq has the advantage that it can be used to filter out mutations in genes that are not expressed in either normal or tumor tissue. This may be desirable since mRNA expression is required to produce proteins with altered or disrupted functionality, and the presence of mutant mRNA is essential for neoantigen production and prediction^11^. In light of this, identifying expressed mutations is important to determine altered proteins/pathways, and RNA sequencing (RNA-seq) is an appropriate approach for this task.

Head and neck squamous cell carcinomas (HNSCC) represent a diverse group of cancers characterized by anatomical, phenotypic, etiological, biological and clinical heterogeneity. HNSCC develops via two primary carcinogenic routes: chemical carcinogenesis through excessive use of tobacco and alcohol or high risk human papillomavirus (HPV)-induced tumorigenesis. Patients with HPV-positive (HPV+) and HPV-negative (HPV−) HNSCCs have distinct clinical behavior and molecular profiles^12–14^, with HPV− cases generally associated with worse outcome^15^. Genomic profiling of HNSCC by The Cancer Genome Atlas (TCGA) and other groups has significantly improved our understanding of HPV-related malignancy. We now appreciate tremendous inter- and intra-tumor heterogeneity among the patients. However, these results are complicated by multiple sites of specimen collection and unbalanced HPV status in the TCGA samples (427 HPV− and 99 HPV + samples)^13,16^. To systematically study the molecular profiles of HNSCC with balanced HPV status and from a single hospital with consistent sample collection and treatment protocols, we ascertained HNSCC patients with untreated oropharynx, oral cavity squamous, or larynx cell carcinoma from an ongoing survivorship cohort at the University of Michigan (UM HNSCC cohort). We comprehensively characterized the tumors from these patients, including mRNA-deep sequencing (mRNA-seq) data^7,16^. Here, we demonstrate the utility and reproducibility of identifying expressed variants via mRNA-seq data, combined with targeted gene sequencing to determine the germline versus somatic mutation status of known cancer and pathway-relevant genes. We show that although we cannot distinguish germline from somatic mutations for most genes, there are several useful analyses we can perform that shed light on carcinogenic mechanisms. We elucidate important pathways that differ by HPV status and are known to be associated with risk of HNSCC.

## Results

### Clinical characteristics

Clinical and demographic characteristics of the University of Michigan (UM) HNSCC cohort (n = 36) are representative of the head and neck cancer patient population in our larger cohort and the United States^17^ (Table 1). Patients were diagnosed at a median age of 57 years (range: 48–87 years) and 72% were male. Fifty-six percent presented in the oropharynx, 39% in the oral cavity and 5% in the larynx. Most patients had stage IV disease (78%) and were former smokers (67%). Exactly half of the tumors were HPV+. Most HPV+ tumors were in the oropharynx (94%) and more likely male (94%), whereas most HPV− tumors were in the oral cavity (71%).

**Table 1:** Demographics of the University of Michigan HNSCC cohort.

	Total	HPV−	HPV+
	36	18	18
**Age**			
Median (std)		56.5 (10.2)	58.5 (7.3)
**Gender**			
Male	26	9	17
Female	10	9	1
**HPV type**			
HPV16	14		14
HPV18	1		1
HPV33	1		1
HPV35	2		2
**Anatomical Site**			
Oropharynx	20	3 (17%)	17 (94%)
Oral Cavity	14	13 (72%)	*1 (6%)*
Larynx	2	2 (11%)	0
Hypopharynx	0	0	0
**Tumor Stage**			
I-II	5	4	1
III	3	1	2
IV	28	13	15
**T stage**			
T1-T2	14	6	8
T3-T4	22	12	10
**N stage**			
N0	10	6	4
N1	2	1	1
N2	17	7	10
N3	7	4	3
**Smoking**			
Never	7	3 (17%)	4 (22%)
Former	23	12 (66%)	11 (61%)
Current	6	3 (17%)	3 (17%)

### Overview of variant call set from UM HNSCC RNA-seq data

**Figure 1:**
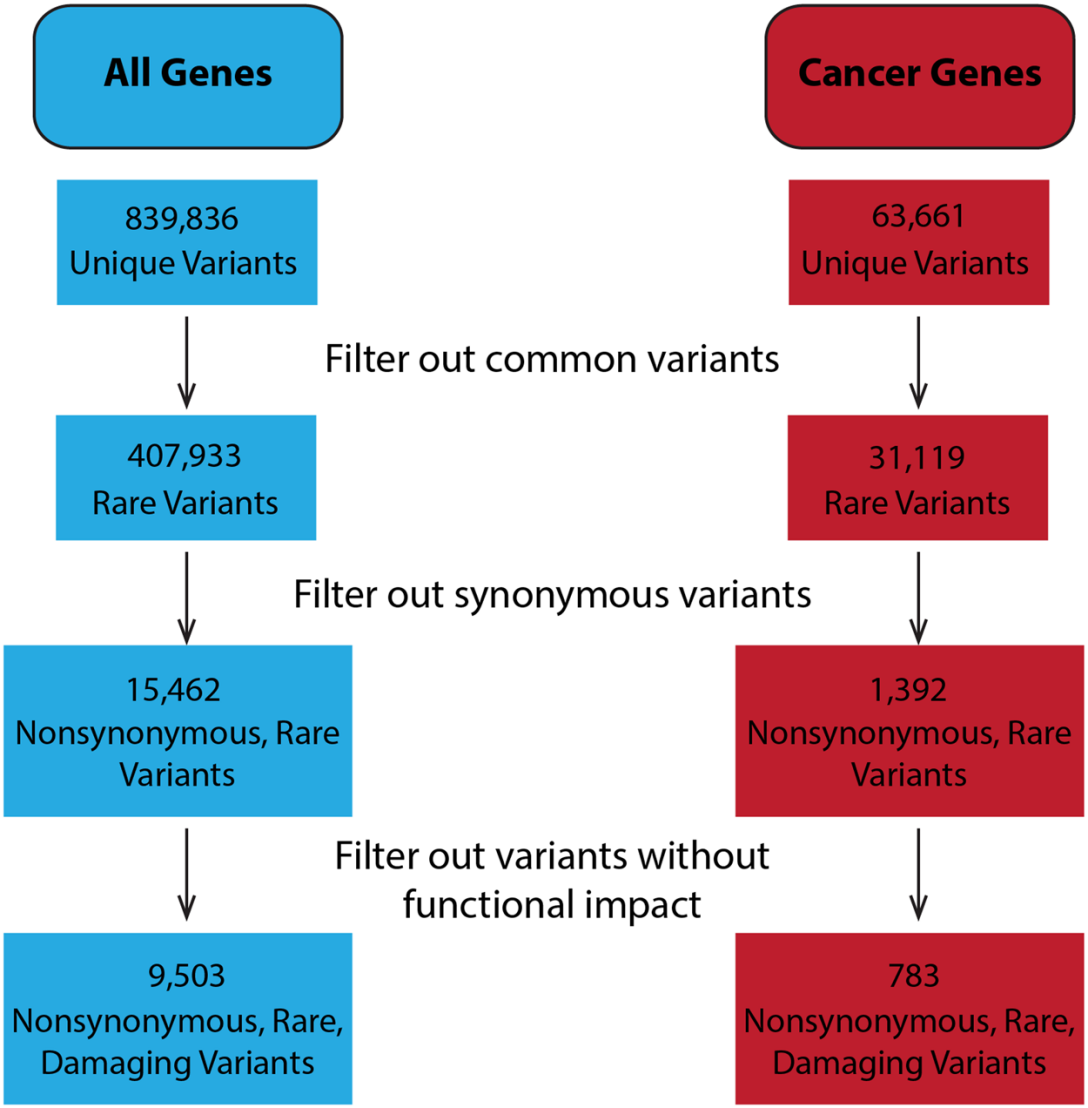
Schematic of the variant filtering steps used to obtain the final numbers of germline and somatic variants across the 36 HNSCC patients in all genes (blue) and in 1344 cancer-related genes defined in methods (red).

Using the GATK best practices pipeline for RNA-seq data, we identified a total of 839,836 germline and somatic variants in the 36 tumor RNA samples (Fig. 1). After filtering out the common variants, 407,933 rare variants remained, of which 15,462 were nonsynonymous. We further refined the identification of candidate functional variants by limiting the variants to those predicted to have deleterious effects on protein function. The final call set contained 9,503 nonsynonymous, rare, and damaging (NRD) single-nucleotide polymorphisms (SNPs) in 6,023 genes, among which 783 SNPs were present in 455 of 1344 identified cancer genes across the tumors (see Methods). The median number of NRD SNPs per sample was 337.5 (Supplementary Figure S1), over 80% of the SNPs were singletons (7816 out of 9503, 82%) and around 92% of them appeared in at most 2 patients. The average transition transversion ratio (Ti/Tv) was 2.05, within the expected range of 2~2.1 when assessing the genome as a whole^18^, and the majority of nucleotide alterations were C > T (G > A).

### Most cancer genes with identified UM RNA NRD variants also harbor somatic mutations identified in the TCGA HNSC cohort

**Figure 2:**
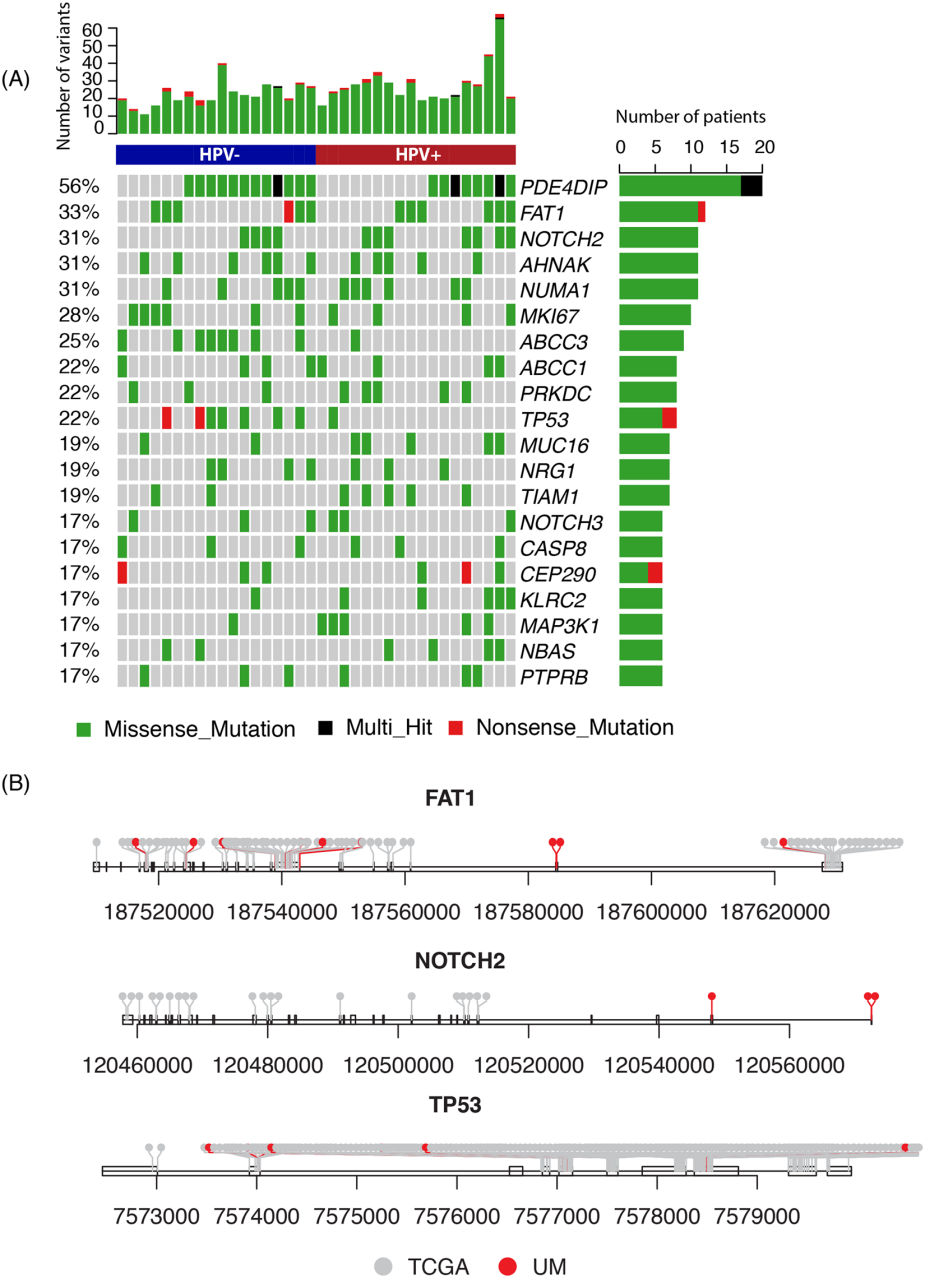
Top 20 frequently mutated cancer genes with both TCGA somatic variants and UM RNA NRD variants. (**A**) Mutation annotation of the top 20 frequently mutated genes in the UM HNSCC cohort. Each row is a gene and each column is a patient sample; (**B**) generic distribution of the variants in three example genes.

To examine whether the identified expressed variants were consistent with the DNA-based findings, we first compared our findings with the somatic mutations identified using WES in the TCGA HNSC cohort. Out of the 455 cancer genes with an identified UM RNA NRD variant (see Methods), 441 of them also harbored a somatic mutation identified in the TCGA (~97%). Figure 2A shows the top 20 mutated cancer genes with both UM RNA NRD variants and TCGA somatic mutations. In the UM sample, these include the gene *FAT1* which was found mutated in 33% (equally distributed among HPV+ and HPV− cases) and is known to be a tumor suppressor gene with somatic mutations in HNSCC as documented in COSMIC database. *NOTCH2*, *TP53*, *NRG1* and *MAP3K1* are also known tumor suppressor genes in multiple cancers and were identified as having recurrent NRD variants in our study. To evaluate the overall concordance between TCGA DNA-based findings and our RNA-seq based results, we extracted the 181 oncogenes and 63 tumor suppressor genes from the COSMIC database and evaluated the number of genes found to carry both TCGA somatic mutations and UM RNA NRD variants. In total, 63 oncogenes were identified to carry at least one UM RNA NRD variant and all of them also carried ≥1 TCGA somatic mutation; 28 tumor suppressor genes were found to carry UM RNA NRD variants and 27 of them also carried ≥1 TCGA somatic mutations (Table 2, Supplementary Table S1). The UM RNA NRD variants were mostly located at the same or close genomic regions as those recorded in TCGA such as is shown for mutations in *FAT1* and *TP53*, with some exceptions in *NOTCH2* where the RNA variants were found at different exons (Fig. 2B).

**Table 2:** Number of oncogenes or tumor suppressors with TCGA somatic mutations and/or UM HNSCC RNA nonsynonymous, rare, and damaging (NRD) mutations.

Gene Type	Total	With UM RNA NRD	With TCGA somatic variants	Overlap	Percentage overlap (%)
Oncogenes	181	63	170	63	100
Tumor suppressors	63	28	62	27	96.43

### Germline versus somatic status of RNA NRD variants identified by targeted gene sequencing

**Figure 3:**
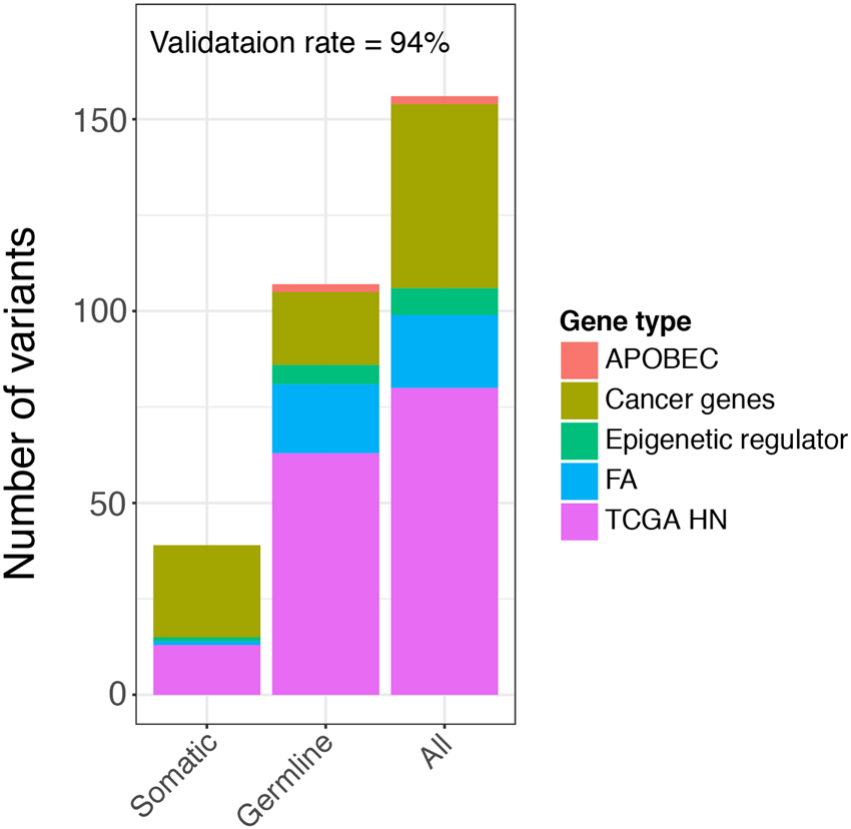
Distribution of the number of validated RNA NRD variants among the different types of genes in the targeted gene panel.

We distinguished germline from somatic mutations by using the targeted gene sequencing data covering 72 genes (see Methods) in tumor and blood paired samples from the same set of patients. Overall, 156 of the RNA NRD variants were located in the target panel genes, out of which 39 were found to be somatic mutations and 107 were determined to be germline mutations in the DNA samples, resulting in a total overlapping rate of 94% (Fig. 3), i.e. 6% of the RNA NRD variants were false positives, ~25% of the variants were somatic and 69% of the variants were germline. Fifty-two (98%) of the 53 genes carrying an identified RNA NRD variant also carry a TCGA somatic mutation (Supplementary Table S2, the column H with column K listed as “yes”). The RNA variants that were not present in the DNA samples included 1 C > A, 1 C > G, 4 C > T, 3 G > A and 1 T > C mutations, not including A > G SNPs that were reported as frequent RNA editing events^9^. For the 72 target panel genes, we examined the germline/somatic ratio with respect to each of the 5 categories of genes chosen for the panel (general cancer-related genes, Fanconi Anemia (FA) genes, APOBEC genes, TCGA HNSC genes and epigenetic regulators). These five categories and the specific genes in each were selected based on the combination of our previous findings and what was already known to be likely relevant to HNSCC carcinogenesis in the literature. We found that the general cancer-related genes had significantly more somatic mutations (24/49; 49%) than all other genes (15/155; 10%), with the odds ratio (OR) of 0.11 (Fisher’s exact test p = 1.92 × 10^−8^), whereas FA genes had only 1/24 somatic mutations and TCGA HN genes had 13/122 somatic mutations, both with significantly more germline mutations (OR = 6.12 and 3.87 respectively, Fisher’s exact test p = 0.05 and 2.47 × 10^−4^). All three APOBEC mutations were germline and 5 out of 6 mutations in epigenetic regulators were germline (Supplementary Table S2). Germline mutations in FA genes have shown evidence for being associated with increased risk of HNSCC^19–21^. Of the 21 germline variants found for FA genes, 2 were not documented in dbSNP database (potentially novel variants) and 3 were annotated as cancer-related by COSMIC database; five were predicted to be benign, 1 in FANCA was likely pathogenic, 5 conflicting or uncertain pathogenicity, and 7 benign/likely benign according to ClinVar clinical significance^22^ (Supplementary Table S3). As supporting evidence that the identified germline variants in FA genes are clinically relevant, we found that patients with an FA germline mutation (FA+) were slightly younger than those without an FA germline mutation (FA−) (average age was 56 years among FA+ vs. 62 years among FA−, t-test p = 0.036).

### RNA NRD variants in FA cancer genes were more abundant in HPV+ patients

Our group and others have shown that HNSCC tumors differ by HPV status in terms of their transcriptome profiles, copy number alterations, and genome-wide DNA methylation and histone modification profiles^7,12,16,23^. We thus examined whether RNA mutational burden differed based on HPV status of the tumor. As expected, the number of called RNA variants was highly correlated with the number of sites covered by the mRNA-seq data (Supplementary Figure S2). Therefore, to adjust for this we applied linear regression using breadth of coverage as a covariate to examine the association between the burden of NRD variants and HPV status. Neither the number of NRD variants overall nor the number of NRD variants in cancer genes significantly differed by HPV status (linear regression, p* = *0.26 and 0.32 respectively). However, we noticed that some FA genes such as *PALB2* had variants only in HPV+ patients, and we found that patients with at least one variant in a cancer-related FA gene were slightly more likely to be HPV+, although not statistically significant (Fisher’s exact test, OR = 4.19, p = 0.08). Cancer-related FA genes were defined as those known to be important in HNSCC or cancer in general, including those carrying recurrent mutations in cancers, having clinical effect when targeted by drugs, or being significantly associated with cancer-related citations in the literature (see Methods). We thus further tested whether the number of NRD variants in the FA pathway differed by HPV status. After adjusting for breadth of coverage, the number of NRD variants in cancer-related FA genes was found to be significantly associated with HPV status (linear regression, coefficient = 0.6, p = 0.02). This is interesting, in light of our previous finding that FA genes are up-regulated in HPV+ patients^16^. These findings suggest that the identified RNA NRD variants in FA genes were associated with carcinogenesis in the HPV+ HNSCC patients.

### Mutagen signature analysis points to the importance of DNA mismatch repair and confirms APOBEC mutations differ by HPV status

**Figure 4:**
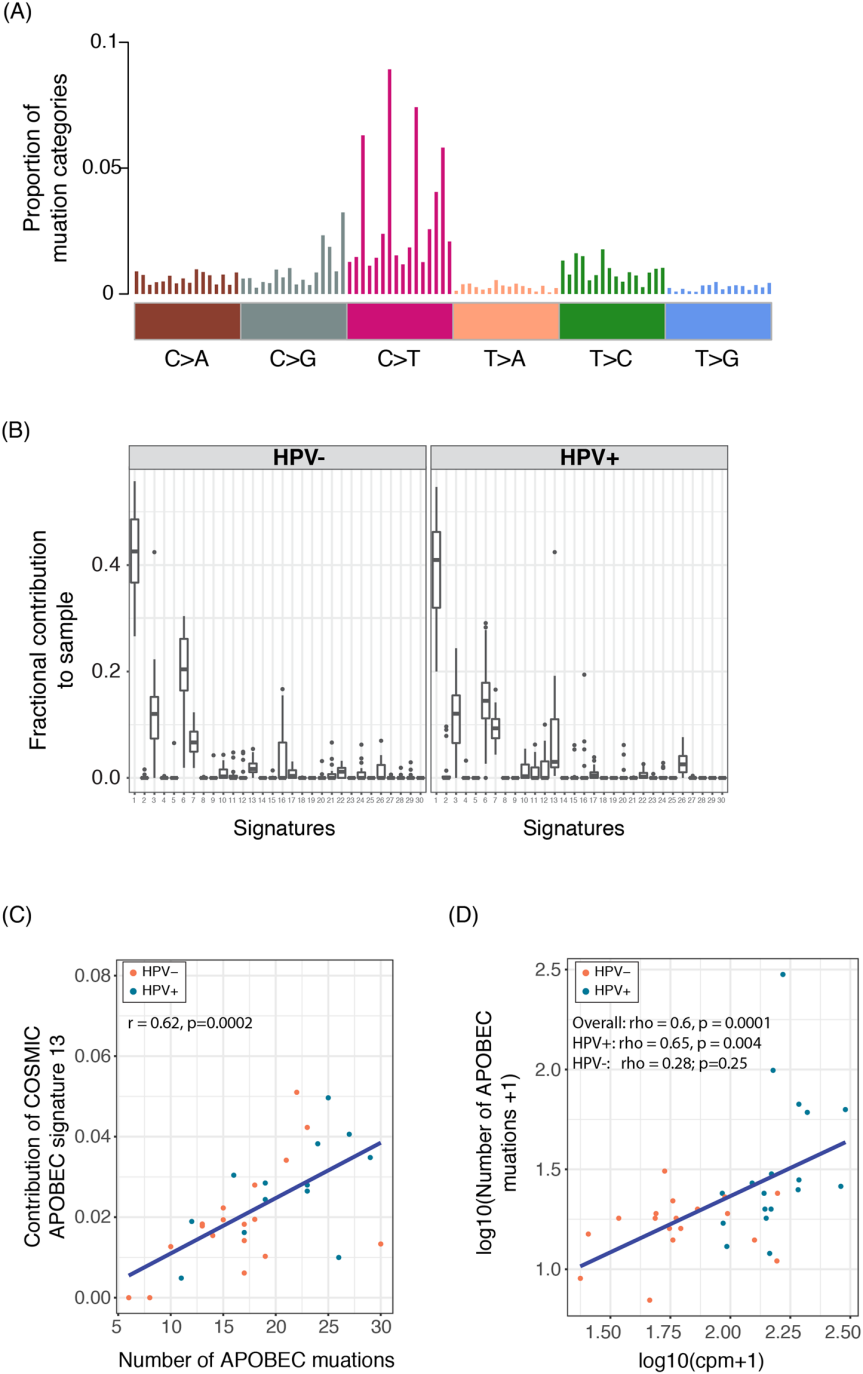
Mutational signature results of the RNA NRD variants: (**A**) distribution of the 96 mutation types combined across patients; each class is divided into 16 categories corresponding to the combinations of bases immediately 5′ and 3′ to each mutation base (context information), and the frequency of each mutation category per sample was computed^24^. (**B**) Fractional contribution of the 30 COSMIC mutational signatures to the combined UM HNSCC tumors by HPV status. See http://cancer.sanger.ac.uk/cosmic/signatures for interpretation of the signatures. (**C**) Correlation between the number of APOBEC-induced mutations and the fractional contribution of the APOBEC signature (#13). (**D**) Correlation between the log combined expression level of APOBEC family genes and the log number of APOBEC-induced mutations.

Mutations found in cancer are the consequence of various biological processes (e.g., age, tobacco exposure, APOBEC-mediated mutations, and UV light). The different combinations of mutation types, i.e. “mutational signatures”, often reflect different mutational processes. Previously the Stratton group identified 30 mutational signatures^24^. We investigated how the predefined mutational signatures are represented in the RNA NRD of the UM HNSCC cohort (Fig. 4A). The matrix decomposition analysis revealed that the top 3 most attributive signatures were: *i)* Signature 1 (median coefficient = 0.41), the result of an endogenous mutational process initiated by spontaneous deamination of 5-methylcytosine, which is correlated with age of cancer of diagnosis; *ii)* Signature 3 (median coefficient = 0.12), which is associated with failure of DNA double-strand break-repair by homologous recombination (DNA-DSBR); and *iii)* Signature 6 (median coefficient = 0.17), that is associated with defective DNA mismatch repair (DNA-MMR) (Fig. 4B). Markedly, the additive contribution of all DNA MMR-related signatures (signature 6, 15, 20 and 26) and DNA-DSBR signature 2 explained on average ~55% (range: 37~73%) of the identified RNA NRD variants in the UM HNSCC patients (Supplementary Figure S3), second only to Signature 1. Taken together, these findings suggest that mutations in DNA MMR and DSBR may be large contributors to HNSCC tumorigenesis, regardless of their germline or somatic status.

On the other hand, we found that Signature 13, which is associated with activity of the AID/APOBEC family of cytidine deaminases converting cytosine to uracil (APOBEC), was significantly more attributive in HPV+ than HPV− patients (fold change = 4.07, Wilcoxon rank-sum test p = 0.02). The contribution of Signature 13 was highly correlated with the direct estimate of the number of APOBEC-induced variants (TCW > T or TCW > G motif, where W is A or T) regardless of HPV status (Pearson’s correlation coefficient = 0.62, p = 0.0002) (Fig. 4C). All of the APOBEC3 family genes were found to be significantly up-regulated in expression in HPV+ patients compared to HPV− ones (edgeR linear model, FDR < 0.05, log2FC ≥ 1)^16^. Also, APOBEC3 family genes were previously identified as being often mutated in multiple cancer types such as bladder, breast, cervix and thyroid cancer and lung adenocarcinoma (LUAD)^25^, and it has been suggested that APOBEC3 proteins link viral infections to cancer development^26^. Moreover, the number of APOBEC-induced variants was identified to be significantly correlated with expression levels of APOBEC family genes at both overall and individual gene level, and this correlation was mainly due to the association in HPV+ patients, suggesting the APOBEC proteins do not play a major role in HPV− mutagenesis (Fig. 4D and Supplementary Figure S4). This is in line with the previous finding that APOBEC-mediated mutagenesis throughout cancer genomes is pervasive and correlates with APOBEC mRNA levels^27,28^. In particular the expression of APOBEC3G and APOBC3C genes were most highly correlated with the APOBEC-associated mutational signature (rho = 0.49 and 0.41, p = 0.002 and 0.014, respectively), and the expression of APOBEC3C was found to be the most attributive to the APOBEC-induced mutational burden (using multi-variate linear regression with all 7 APOBEC genes, p = 0.007; Supplementary Table S4). In addition, cancer-related FA + patients tend to be associated with higher APOBEC signature than FA- patients (coefficient ratio = 2.34, Wilcoxon rank-sum test p = 0.03), indicating the relatedness between the APOBEC proteins and FA/DNA repair pathways.

## Discussion

Most analyses of mutations in cancer focus only on somatic alterations. However, germline mutations are also important for cancer initiation and progression, including interplaying with the somatic mutations, which is often overlooked. Using mRNA-seq data from 36 University of Michigan HNSCC patients (18 HPV+ and 18 HPV−), we identified expressed germline and somatic variants that were rare and predicted to potentially have deleterious effects on the encoded proteins. We demonstrated that the majority of cancer genes harboring these variants were documented in the somatic mutation profile of HNSCC patients by TCGA. Many of the findings of TCGA using WES were also found in our study using mRNA-seq, including the APOBEC mutational signature in HPV+ patients. We complemented the mRNA-seq analysis with targeted gene sequencing data to determine germline versus somatic mutation status for a select set of 72 genes. Since HPV status is closely associated with the genomic landscape in HNSCC, we took advantage of the balanced HPV status in the UM HNSCC cohort and focused on the comparison of genetic profiles between HPV+ and HPV− patients. Our results reproduced known differences: that HPV− HNSCC inactivates the cell cycle suppressor TP53 (7 HPV− vs. 1 HPV+ HNSCCs harbor variants) and CDKN2A (4 HPV− vs. 2 HPV+ HNSCCs harbor variants) by nonsynonymous single nucleotide polymorphism^13^.

The FA pathway coordinates elements of three basic DNA repair pathways including homologous recombination (HR), nucleotide excision repair (NER), and mutagenic translesion synthesis (TLS)^29^, and it has been shown that crosstalk exists between the FA and mismatch repair (MMR) pathways^30^. The number of expressed NRD variants in cancer-related FA genes was significantly higher in HPV+ patients, and patients with an FA variant were slightly younger than those with no FA gene variants (average age was 56 years among FA + vs. 62 years among FA-, t-test p = 0.036). The expressed NRD variants identified in FA genes were mostly found to be germline mutations by the targeted gene sequencing, suggesting a genetic predisposition to HNSCC. In fact, FA genes with biallelic germline mutations are associated with a 700-fold increased risk of HNSCC^31,32^, with the most common site being the oral cavity. Based on our results we speculate that a heterozygous, germline FA mutation combined with a second risk factor, such as HPV infection or smoking, may increase the risk of HNSCC and at a slightly younger age. This hypothesis will need to be tested in a larger cohort with additional evidence to support the pathogenicity of their FA variants. One reason our study may have been able to detect a significant difference in age is due to our focus on oral cavity and oropharyngeal tumors, and our wide range of ages at diagnosis. In addition, the mutation status in cancer-related FA genes was found to differentiate the contribution of the APOBEC mutagen signature to the mutational distribution in HNSCC, consistent with previous studies showing that APOBEC proteins promote the cytidine deaminase-dependent DNA repair process and are involved in mutational double-strand DNA break repair following ionizing radiation treatments^33,34^. Previous analysis of TCGA data also detected evidence that HPV infection was strongly associated with APOBEC-mediated mutagenesis in HNSCC^35^. These findings point to the general importance of Fanconi Anemia and DNA repair in HNSCC pathogenesis and highlight the importance of integrating population studies of risk with somatic mutational analyses.

Comprehensive characterization of the 36 patient UM HNSCC cohort has allowed our group to define important genetic and epigenetic characteristics of HNSCC especially for HPV-associated tumors, resulting in several novel findings: *i)* two subtypes of HPV+ HNSCC were identified with distinct transcriptional profiles, which were associated with HPV characteristics, specific CNAs, PIK3CA mutations and pathway signatures^16^; *ii)* HPV integration in HNSCC was associated with worse survival outcomes, weakened immune response signatures and suggested novel candidate drivers^7^. The current work complements the previous studies from the perspective of genetic variation, providing a more comprehensive view of the molecular characteristics of this cohort on which future studies can be based.

While RNA-based variant calling may miss some important mutations that are not transcribed, it is a feasible approach to detecting expressed mutations given the large amount of existing RNA-seq data in cancer studies. Many studies have shown the value of using RNA-seq data to study genetic variants^36,37^. Although calling genetic variants from RNA-seq has been controversial due to the intrinsic complexity of transcriptomes and limitations in variant calling algorithms, significant effort has resulted in improvements to the analysis pipeline^38–40^. The GATK best practice of RNA-seq variant calling pipeline, a currently widely applied pipeline, effectively addresses the issue of mapping the alternative spliced transcripts to the reference genome by the STAR 2-pass approach and Split ‘N’ Trim technique^41^. To control the false positives and potential RNA editing events in our call set, we imposed a series of vigorous filtering, and limited the variants to nonsynonymous, rare and damaging ones in cancer-related genes, over 90% of which were successfully validated by the targeted gene sequencing. Since the NRD mutational burden inferred from RNA-seq is highly correlated to the number of covered bases, we introduced breadth of coverage as a covariate in linear models to investigate the relationship between NRD mutational burden and HPV status. One limitation of our study is the lack of genome-wide germline data. After applying filters to focus on damaging variants the final call set contained 73% germline mutations as indicated by the germline data for the 72 target panel genes. However, the proportion of somatic and germline mutations depends on the type of genes, and indeed cancer genes were found to harbor significantly more somatic mutations.

In conclusion, we used mRNA-seq data generated from the UM HNSCC cohort to identify expressed germline and somatic deleterious variants, and we validated the results by targeted gene sequencing on both somatic and germline samples. Although our study was limited by only being able to distinguish germline from somatic mutations for 72 genes of interest, we show that valid and useful conclusions can be made with combined NRD germline and somatic variants. The results recapitulate many TCGA findings and highlight the association between FA/DNA repair pathways and HPV status in HNSCC tumorigenesis, which complement our previous studies based on the same cohort. Based on this one-center high-quality HNSCC cohort, we have analyzed multi-dimensional molecular profiles that we hope will be valuable for future studies in HNSCC and other cancer sites.

## Methods

### Recruitment and tissue ascertainment

From 2011 to 2013, incident, previously untreated HNSCC patients with initial diagnosis of oropharynx, oral cavity or larynx tumors were screened for eligibility and approached to sign a written, informed consent for collection of tumor tissue and whole blood. Written informed consent was obtained from all patients, and the study was approved by the University of Michigan Institutional Review Board. The IRB Authorization Agreement has been approved. Tumor tissue and blood were immediately placed into a cryogenic storage tube and flash frozen in liquid nitrogen by surgical staff in the hospital procedures unit. Flash frozen tissue specimens were transported in liquid nitrogen and stored at −80 °C until prepared for histology. The frozen tissues were embedded in OCT media in vinyl cryomolds on dry ice and stored in −80 °C. H&E slides were sectioned from frozen tissue on a cryostat for each tumor specimen and assessed by a board-certified pathologist from the Pathology Department of the University of Michigan and assessed for degrees of cellularity and necrosis. Specimens exhibiting 70% or greater cellularity and less than 10% necrosis were selected for further study. In total, 36 patients were included in this study. All experimental protocols used to process the specimens were approved by University of Michigan Institutional Review Board, and all methods were carried out in accordance with relevant guidelines and regulations.

### RNA library preparation and sequencing

Surface scrapings from the region of tissue identified as having 70% or greater cellularity were taken directly from the frozen tissue block using a sterile scalpel. Processing was performed over dry ice without allowing the tissue to thaw and frozen scrapings were placed into pre-chilled tubes on dry ice. Tissue scrapings were processed using the Qiagen AllPrep DNA/RNA/Protein Mini Kit (Valencia, CA, USA) as per manufacturer protocol. DNA and RNA concentration and quality were assessed using a Nanodrop 2000 spectrophotometer (Marietta, OH, USA). RNA quality and concentration was also assessed by Agilent Bioanalyzer. Whole blood DNA from the same patients was isolated using the Qiagen QIAamp Blood DNA Mini Kit (Valencia, CA, USA), according to manufacturer protocol. RNA library construction and sequencing on Illumina HiSeq (Valencia, CA, USA) using 100 nucleotide paired-end reads were performed by the University of Michigan DNA sequencing Core Facility (GSE74956, details see our previous work^16^).

### Determination of HPV status and type

Each of the 36 tumors were previously classified as either HPV-positive or HPV-negative using the RNA-seq data, as described in^16^. Briefly, reads were aligned to the reference genomes for high risk HPV types 16, 18, 31, 33, 35, 39, 45, 51, 52, 56, 58, 59, 66 and 68, and those having > 500 reads were classified as HPV+. Among the 36 samples, 14 were type HPV-16, one was HPV-18, one was HPV-33, and two were HPV-35.

### RNA variant calling and annotation

Variant calling was performed by following GATK Best Practices for RNA-seq data to identify single nucleotide polymorphisms (SNPs) and small indels (https://software.broadinstitute.org/gatk//guide/article?id=3891). Briefly, paired-end reads were aligned to the human genome hg19 using STAR 2-pass^42^ and duplications were marked by Picard (http://broadinstitute.github.io/picard/). GATK was used for indel realignment and base recalibration. Variants were called for multiple samples using HaplotypeCaller (https://software.broadinstitute.org/gatk/documentation/tooldocs/current/org_broadinstitute_gatk_tools_walkers_haplotypecaller_HaplotypeCaller.php). Variants which fell under any of the following criteria were filtered out: quality scores less than 25 (QUAL < 25), read depth less than 10 (DP < 10), strong strand bias (FS > 30), normalized quality score less than 2 (QD < 2.0), or variants part of a SNP cluster that defined as 2 SNPs within 35 bps indicating a false positive. To identify nonsynonymous, rare and damaging (NRD) variants, resulting variants were filtered by three steps: *i)* the variants predicted to disrupt the primary structure of the protein, i.e. nonsynonymous variants, were identified by SnpEff^43^; *ii)* rare variants defined as variants that are present in less than 5% of 1000 Genomes^44^ and NHLBI Exome Sequencing Project^45^ subjects annotated by ANNOVAR^46^; *iii)* damaging variants were identified by either PolyPhen v2^47^ or SIFT^48^.

### Definition of cancer genes

To further prioritize cancer-associated mutations, we created a list of cancer-related genes derived from several public sources. We included genes that met any of the following criteria: 1) the gene was reported to harbor significant or recurrent mutations in head and neck cancer^2,13,49^ (212 genes), 2) the gene was included in the Catalog of Somatic Mutations in Cancer (COSMIC), a large database of cancer somatic gene mutations curated by the Wellcome Trust Sanger Institute^50^ (547 genes), 3) the gene qualified as an “actionable target” or “pharmacogenetic target” in cancer by Wagle and colleagues^51^ (137 genes), 4) the gene was significantly often associated with the Medical Subject Heading (MeSH) terms “stem cell” or containing the keyword “neoplasm” in biomedical literature (using Gene2MeSH^52,53^; 308 genes) or 5) the gene was defined as a “Mut-driver” by Vogelstein and colleagues^54^ (140 genes). In total, 1344 genes met this criteria and were defined as cancer genes in this study.

### TCGA data comparison

Somatic mutation annotation format (MAF) file (version 2.4) generated by Canada’s Michael Smith Genome Sciences Center (bcgsc.ca) was downloaded from the TCGA data portal (https://portal.gdc.cancer.gov), and compared with our called RNA NRD variants. The frequency of the genes that harbor both University of Michigan RNA NRD and TCGA DNA somatic mutations were reported.

### Targeted gene sequencing and variant calling

A panel of 72 genes were selected for validation by targeted DNA sequencing. Out of the 72 gene panel, 12 were classified as general cancer-related genes, 14 were FA genes, 6 were APOBEC genes, 36 were TCGA head and neck cancer genes, and 4 were epigenetic regulator genes. In order to generate mutually exclusive gene groups for presentation, we prioritized them from highest to lowest in the following order: FA, APOBEC, Epigenetic regulators, cancer-related and TCGA HN genes (Supplementary Table S5). The targeted sequencing was performed on the 36 matched tumor and blood DNA samples from the same cohort. Capture of the target regions (exons plus splice junctions) was carried out using a custom-designed NimbleGen SeqCap Target Enrichment kit (Roche) per manufacturer protocols. The DNA libraries were prepared using the SeqCap EZ Choice Library Kit. Libraries were sequenced using 125 bp paired-end reads on Illumina HiSeq. 2500 at the UM DNA Sequencing Core. The variants were called following the GATK Best Practices pipeline: (*i*) germline mutations were called by HaplotypeCaller GVCF pipeline (https://software.broadinstitute.org/gatk/best-practices/bp_3step.php?case=GermShortWGS), and (*ii*) somatic mutations were called using the Mutect2 pipeline (https://software.broadinstitute.org/gatk/best-practices/mutect2.php).

### Determining the relationship between RNA NRD mutational burden in FA cancer genes and HPV status

The FA pathway is comprised of approximately 17 genes^55^, 10 of which have been shown to be associated with various cancers: *FANCA*, *FANCC*, *FANCD1* (*BRCA2*), *FANCD2*, *FANCE*, *FANCF*, *FANCG*, *FANCJ* (*BRIP1*), *FANCN* (*PALB2*) and *FANCS* (*BRCA1*)^56^. Out of the 17 FA genes, 2 genes that do not contain any NRD variants were excluded from this study (*RAD51C* and *XPF*). The patients were categorized by FA gene mutation status, i.e. the patients with a germline or somatic mutation in at least one of the FA genes were considered as FA + , and the rest were FA−. To compare the RNA NRD mutational burden by different conditions, a linear regression model was used to model the number of NRD mutations in the 10 cancer-related FA genes as a function of HPV status, using the breadth of coverage as a covariate, i.e. the number of ≥10x covered sites.

### Identification of the mutagen signatures contributing to the NRD variants

The NRD variants in each patient were categorized into one of the 96 possible categories: 6 classes of base substitution (C > A, C > G, C > T, T > A, T > C and T > G) × 16 combinations of bases immediately 5′ and 3′ to each mutation base (context information), and the frequency of each mutation category per sample was computed^24^. The previously defined 30 mutational signatures were downloaded from COSMIC (http://cancer.sanger.ac.uk/cosmic/signatures). Assuming the mutational distribution of a single sample is a linear combination of the known 30 signatures, we used non-negative least squares (NNLS) method^57^ to decompose the mutational signatures (a 96 × 30 matrix) for the observed mutational distribution of each HNSCC patient (a 96 × 1 vector). The difference in the contribution (coefficient) of each known mutational signature by HPV or FA status was accessed by Wilcoxon rank-sum test with Benjamini-Hochberg correction.

### Determining the relationship between APOBEC gene expression and APOBEC-induced mutational burden

We chose the stringent TCW motif (where W corresponds to either A or T) to represent the APOBEC-induced mutational patterns and the mutations include C > T and C > G (TCW to TTW or TGW and the complementary WGA to WAA or WCA) changes^27^. The number of NRD mutations with any of the above patterns was summarized for each patient, representing the APOBEC-induced mutational burden. The expression levels (count per million, CPM) of all APOBEC family genes were extracted from the mRNA-seq data generated from the same set of cohort^16^, and 7 APOBEC genes (*APOBEC2*, *APOBEC3A*, *APOBEC3B*, *APOBEC3C*, *APOBEC3D*, *APOBEC3F* and *APOBEC3G*) that have average CPM level greater than 1 were included in the analysis. The correlation between the expression level of individual APOBEC genes (or combined APOBEC genes) and the APOBEC-induced mutational burden were evaluated by Spearman correlation test. To identify the specific APOBEC genes whose expression levels significantly contribute to the APOBEC-induced mutations, a linear regression model was applied to assess the relationship between the individual gene’s expression levels and the APOBEC mutational burden.

### Data availability

All data has been deposited in the Gene expression Omnibus (GEO) under accession number: GSE74956 (RNA-seq) and SRP148108 (targeted gene sequencing).

## Electronic supplementary material

Supplementary Material

Table S1

Table S2

Table S3

Table S4

Table S5

## References

[1] Chapman MA (2011). Initial genome sequencing and analysis of multiple myeloma. Nature.

[2] Stransky N (2011). The mutational landscape of head and neck squamous cell carcinoma. Science.

[3] Pickrell JK (2010). Understanding mechanisms underlying human gene expression variation with RNA sequencing. Nature.

[4] Rozowsky J (2011). AlleleSeq: analysis of allele-specific expression and binding in a network framework. Mol Syst Biol.

[5] Montgomery SB (2010). Transcriptome genetics using second generation sequencing in a Caucasian population. Nature.

[6] Liu J (2012). Genome and transcriptome sequencing of lung cancers reveal diverse mutational and splicing events. Genome Res.

[7] Koneva, L.A. *et al*. HPV Integration in HNSCC Correlates with Survival Outcomes, Immune Response Signatures, and Candidate Drivers. *Mol Cancer Res* (2017). 10.1158/1541-7786.MCR-17-0153 PMC5752568 28928286

[8] Bahn JH (2012). Accurate identification of A-to-I RNA editing in human by transcriptome sequencing. Genome Res.

[9] Ramaswami G (2013). Identifying RNA editing sites using RNA sequencing data alone. Nat Methods.

[10] Ramaswami G (2012). Accurate identification of human Alu and non-Alu RNA editing sites. Nat Methods.

[11] Karasaki T (2017). Prediction and prioritization of neoantigens: integration of RNA sequencing data with whole-exome sequencing. Cancer Sci.

[12] Sartor MA (2011). Genome-wide methylation and expression differences in HPV(+) and HPV(−) squamous cell carcinoma cell lines are consistent with divergent mechanisms of carcinogenesis. Epigenetics.

[13] Cancer Genome Atlas N (2015). Comprehensive genomic characterization of head and neck squamous cell carcinomas. Nature.

[14] Chakravarthy A (2016). Human Papillomavirus Drives Tumor Development Throughout the Head and Neck: Improved Prognosis Is Associated With an Immune Response Largely Restricted to the Oropharynx. J Clin Oncol.

[15] Taberna M (2017). Human papillomavirus-related oropharyngeal cancer. Ann Oncol.

[16] Zhang Y (2016). Subtypes of HPV-Positive Head and Neck Cancers Are Associated with HPV Characteristics, Copy Number Alterations, PIK3CA Mutation, and Pathway Signatures. Clin Cancer Res.

[17] Papagerakis S (2014). Proton pump inhibitors and histamine 2 blockers are associated with improved overall survival in patients with head and neck squamous carcinoma. Cancer Prev Res (Phila).

[18] Genomes Project C (2015). A global reference for human genetic variation. Nature.

[19] Kutler DI (2003). High incidence of head and neck squamous cell carcinoma in patients with Fanconi anemia. Arch Otolaryngol Head Neck Surg.

[20] Levitus M, Joenje H, de Winter JP (2006). The Fanconi anemia pathway of genomic maintenance. Cell Oncol.

[21] Masserot C (2008). Head and neck squamous cell carcinoma in 13 patients with Fanconi anemia after hematopoietic stem cell transplantation. Cancer.

[22] Landrum MJ (2018). ClinVar: improving access to variant interpretations and supporting evidence. Nucleic Acids Res.

[23] Zhang Y, Lin YH, Johnson TD, Rozek LS, Sartor MA (2014). PePr: a peak-calling prioritization pipeline to identify consistent or differential peaks from replicated ChIP-Seq data. Bioinformatics.

[24] Alexandrov LB (2013). Signatures of mutational processes in human cancer. Nature.

[25] Rebhandl S, Huemer M, Greil R, Geisberger R (2015). AID/APOBEC deaminases and cancer. Oncoscience.

[26] Downey RF (2015). Human endogenous retrovirus K and cancer: Innocent bystander or tumorigenic accomplice?. Int J Cancer.

[27] Roberts SA (2013). An APOBEC cytidine deaminase mutagenesis pattern is widespread in human cancers. Nat Genet.

[28] Faden DL (2017). Multi-modality analysis supports APOBEC as a major source of mutations in head and neck squamous cell carcinoma. Oral Oncol.

[29] Moldovan GL, D’Andrea AD (2009). How the fanconi anemia pathway guards the genome. Annu Rev Genet.

[30] Peng M, Xie J, Ucher A, Stavnezer J, Cantor SB (2014). Crosstalk between BRCA-Fanconi anemia and mismatch repair pathways prevents MSH2-dependent aberrant DNA damage responses. EMBO J.

[31] Scheckenbach K, Wagenmann M, Freund M, Schipper J, Hanenberg H (2012). Squamous cell carcinomas of the head and neck in Fanconi anemia: risk, prevention, therapy, and the need for guidelines. Klin Padiatr.

[32] Kutler DI (2003). A 20-year perspective on the International Fanconi Anemia Registry (IFAR). Blood.

[33] Nowarski R, Kotler M (2013). APOBEC3 cytidine deaminases in double-strand DNA break repair and cancer promotion. Cancer Res.

[34] Nowarski R (2012). APOBEC3G enhances lymphoma cell radioresistance by promoting cytidine deaminase-dependent DNA repair. Blood.

[35] Henderson S, Chakravarthy A, Su X, Boshoff C, Fenton TR (2014). APOBEC-mediated cytosine deamination links PIK3CA helical domain mutations to human papillomavirus-driven tumor development. Cell Rep.

[36] Sheng Q, Zhao S, Li CI, Shyr Y, Guo Y (2016). Practicability of detecting somatic point mutation from RNA high throughput sequencing data. Genomics.

[37] Coudray, A. B., A. M., Bucher, P., Iyer, VR Detection and benchmarking of somatic mutations in cancer genomes using RNA-seq data. *BioRxiv 249219* [Preprint] (2018). 10.7717/peerj.5362 PMC6074801 30083469

[38] Shah SP (2009). Mutational evolution in a lobular breast tumour profiled at single nucleotide resolution. Nature.

[39] Kridel R (2012). Whole transcriptome sequencing reveals recurrent NOTCH1 mutations in mantle cell lymphoma. Blood.

[40] Piskol R, Ramaswami G, Li JB (2013). Reliable identification of genomic variants from RNA-seq data. Am J Hum Genet.

[41] Institute, B., GATK Best Practices workflow for SNP and indel calling on RNAseq data, Available at https://software.broadinstitute.org/gatk/documentation/article.php?id=3891s.

[42] Dobin A, Gingeras TR (2015). Mapping RNA-seq Reads with STAR. Curr Protoc Bioinformatics.

[43] Cingolani P (2012). A program for annotating and predicting the effects of single nucleotide polymorphisms, SnpEff: SNPs in the genome of Drosophila melanogaster strainw1118; iso-2; iso-3. Fly (Austin).

[44] Genomes Project C (2010). A map of human genome variation from population-scale sequencing. Nature.

[45] Fu W (2013). Analysis of 6,515 exomes reveals the recent origin of most human protein-coding variants. Nature.

[46] Wang K, Li M, Hakonarson H (2010). ANNOVAR: functional annotation of genetic variants from high-throughput sequencing data. Nucleic Acids Res.

[47] Adzhubei, I., Jordan, D. M. & Sunyaev, S. R. Predicting functional effect of human missense mutations using PolyPhen-2. *Curr Protoc Hum Gene*t **Chapter 7**, Unit720 (2013). 10.1002/0471142905.hg0720s76 PMC4480630 23315928

[48] Ng PC, Henikoff S (2003). SIFT: Predicting amino acid changes that affect protein function. Nucleic Acids Res.

[49] Agrawal N (2011). Exome sequencing of head and neck squamous cell carcinoma reveals inactivating mutations in NOTCH1. Science.

[50] Forbes SA (2015). COSMIC: exploring the world’s knowledge of somatic mutations in human cancer. Nucleic Acids Res.

[51] Wagle N (2012). High-throughput detection of actionable genomic alterations in clinical tumor samples by targeted, massively parallel sequencing. Cancer Discov.

[52] Ade AW, Z.S., DJ, Gene2MeSH, Available at http://gene2mesh.ncibi.org, (Mar 2007).

[53] Sartor MA (2010). ConceptGen: a gene set enrichment and gene set relation mapping tool. Bioinformatics.

[54] Vogelstein B (2013). Cancer genome landscapes. Science.

[55] Fanconi Anemia Research Fund, I., *Fanconi Anemia: Guidelines for Diagnosis and Management*. (SciScripter, LLC, 2014).

[56] Futreal PA (2004). A census of human cancer genes. Nat Rev Cancer.

[57] Stokkum, K. M. M. A.I.H.M.v., nnls: The Lawson-Hanson algorithm for non-negative least squares (NNLS), Available at https://cran.r-project.org/web/packages/nnls/index.html (2012).

