# Supplementary material for: Expressed HNSCC variants by HPV-status in a well-characterized Michigan cohort
Source: Sci Rep. 2018 Jul 30;8:11458. doi: 10.1038/s41598-018-29599-w (PMC6065423; doi:10.1038/s41598-018-29599-w)
Supplement: Supplementary file 1 — Supplementary Material [file 41598_2018_29599_MOESM1_ESM.pdf]

Expressed HNSCC variants by HPV-status in a well-characterized Michigan cohort

Tingting Qin[1], Yanxiao Zhang[1,X], Katie R. Zarins[2], Tamara Jones[2], Shama Virani[1,2], Lisa A. Peterson[3], Jonathan B. McHugh[4], Douglas Chepeha[3,§], Gregory T. Wolf[3], Laura S Rozek[2,3*], Maureen A Sartor[1*]

[1]Department of Computational Medicine and Bioinformatics, University of Michigan, Ann Arbor, Michigan, United States of America

[2]Department of Environmental Health Sciences, University of Michigan School of Public Health, Ann Arbor, Michigan, United States of America

[3]Department of Otolaryngology-Head and Neck Surgery, University of Michigan Medical School, Ann Arbor, Michigan, United States of America

[4]Department of Pathology, University of Michigan Medical School, Ann Arbor, Michigan, United States of America

[X]Current address: Ludwig Institute for Cancer Research, 9500 Gilman Drive, La Jolla, CA 92093

[§]Current address: Department of Otolaryngology, University of Toronto, Toronto, Ontario, Canada

*Correspondence/reprints addressed to:

Maureen Sartor

100 Washtenaw Avenue

Computational Med & Bioinformatics

2044 Palmer Commons

Ann Arbor MI 48109-2218

734-763-8013

OR

Laura S. Rozek, Ph.D.

M6529 SPH II

1415 Washington Heights

Ann Arbor, Michigan 48109-2029

Supplementary data

Figure S1. Statistics of the RNA NRD variants identified in the UM HNSCC cohort: (A) distribution of NRD variant burden in each of the 36 samples; (B) distribution of the type of SNPs; (C) number of transition and transversion SNPs.

Figure S2. The mutational burden is highly correlated with breadth of coverage in the RNA-seq data. (A) The total number of RNA variants called by the GATK pipeline is correlated with the number of bases covered by at least 10x reads in the RNA-seq data; (B) the number of RNA NRD variants is also correlated with the number of the bases covered by at least 10x reads in RNA-seq data, but to a lesser extent.

Figure S3. The combined contribution of MMR-related signatures (signature 26 in red color; combined contributions of the original signature 6, 15, 20 and 26) and DBBR signatures (signature 2 in green color) explained more than half of RNA NRD mutations.

Figure S4. Correlations between the gene expression levels and APOBEC-induced mutational burden for seven individual APOBEC genes.

Table S1. The TCGA validated RNA NRD variants in 63 oncogenes and 28 tumor suppressor genes annotated by COSMIC.

Table S2. The RNA NRD variants validated by targeted gene sequencing.

Table S3. The annotation of RNA NRD variants in FA genes.

Table S4. The association between the expression of individual APOBEC gene and the APOBEC-induced mutational burden accessed by a linear regression model.

Table S5. The 72 genes in the targeted gene panel.

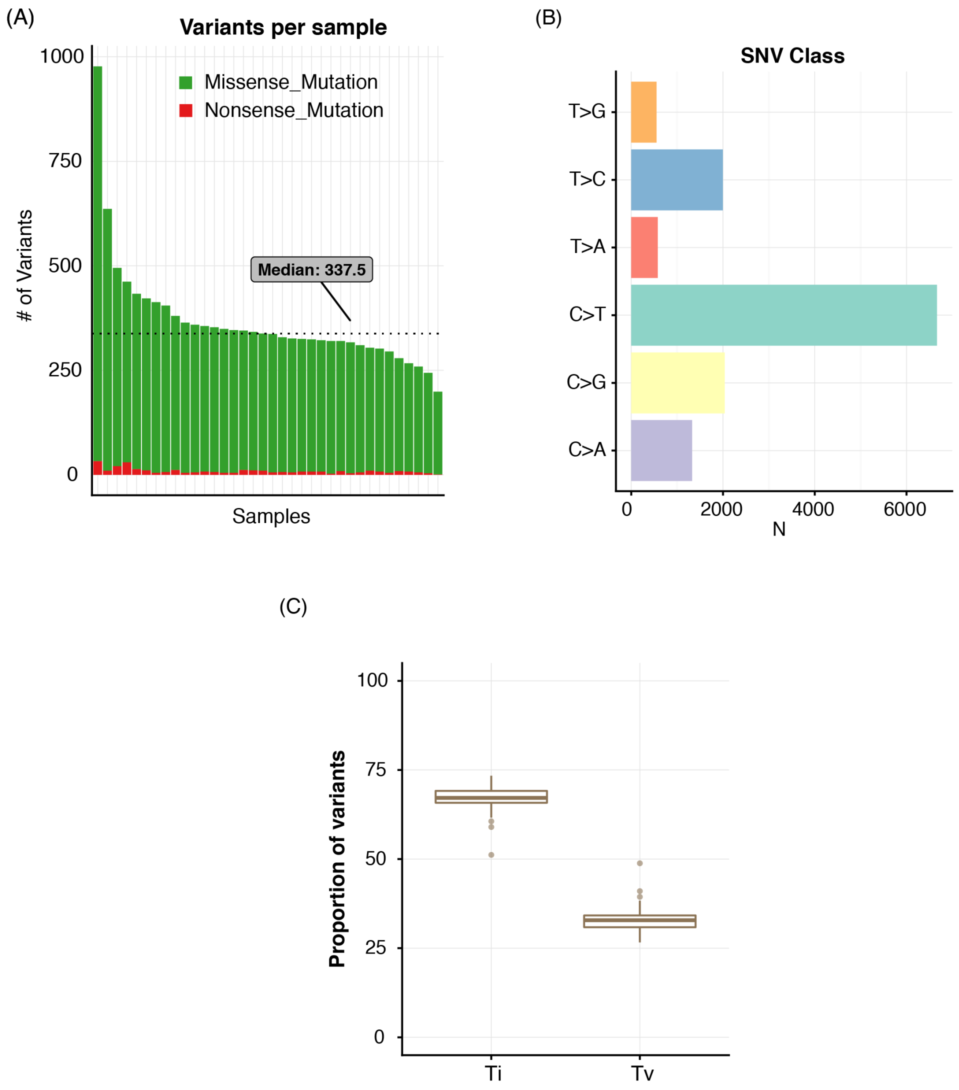
(A)
Variants per sample
Missense_Mutation
Nonsense_Mutation
Median: 337.5
# of Variants
1000
750
500
250
0
Samples
(B)
SNV Class
T>G
T>C
T>A
C>T
C>G
C>A
0
2000
4000
6000
N
(C)
Proportion of variants
100
75
50
25
0
Ti
Tv

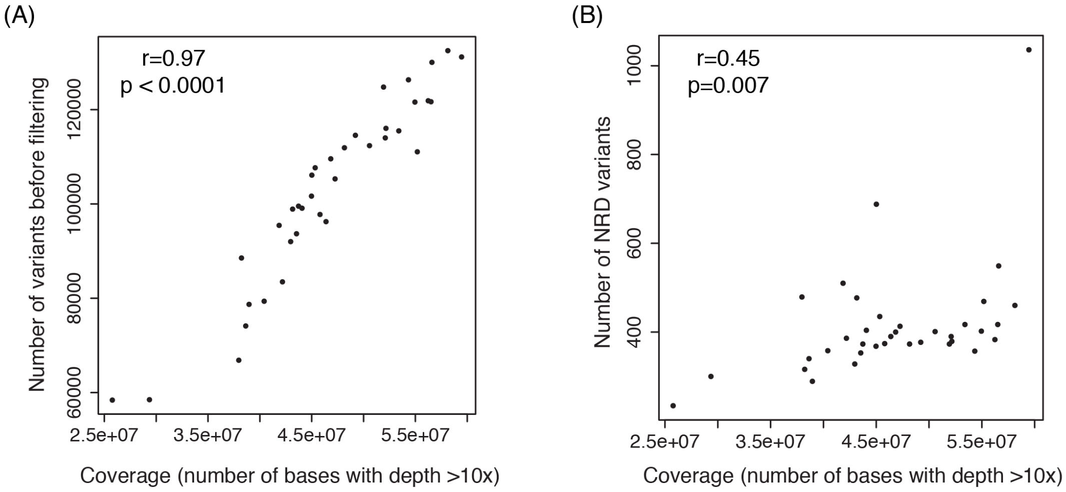
(A)
r=0.97
p < 0.0001
Number of variants before filtering
60000
80000
100000
120000
2.5e+07
3.5e+07
4.5e+07
5.5e+07
Coverage (number of bases with depth >10x)
(B)
r=0.45
p=0.007
Number of NRD variants
400
600
800
1000
2.5e+07
3.5e+07
4.5e+07
5.5e+07
Coverage (number of bases with depth >10x)

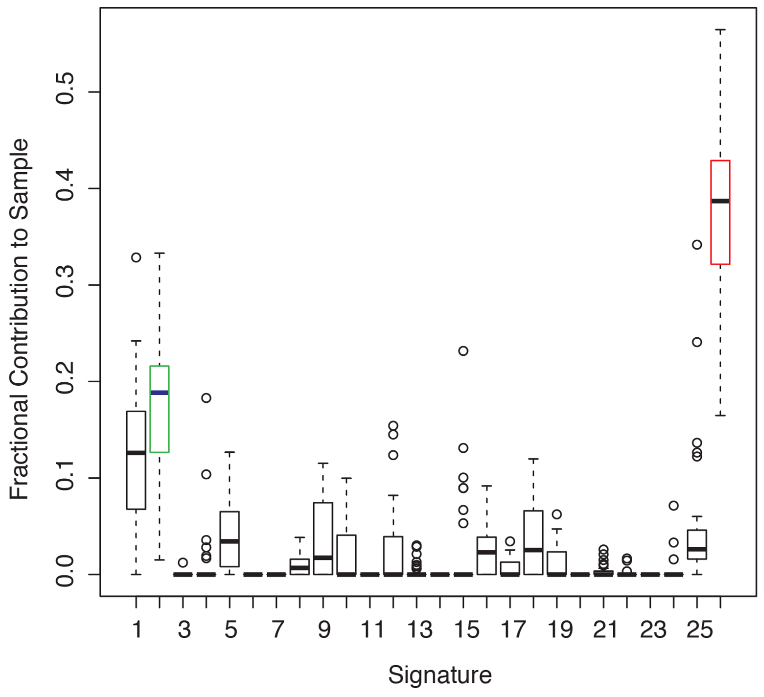
Fractional Contribution to Sample
0.0
0.1
0.2
0.3
0.4
0.5
1
3
5
7
9
11
13
15
17
19
21
23
25
Signature

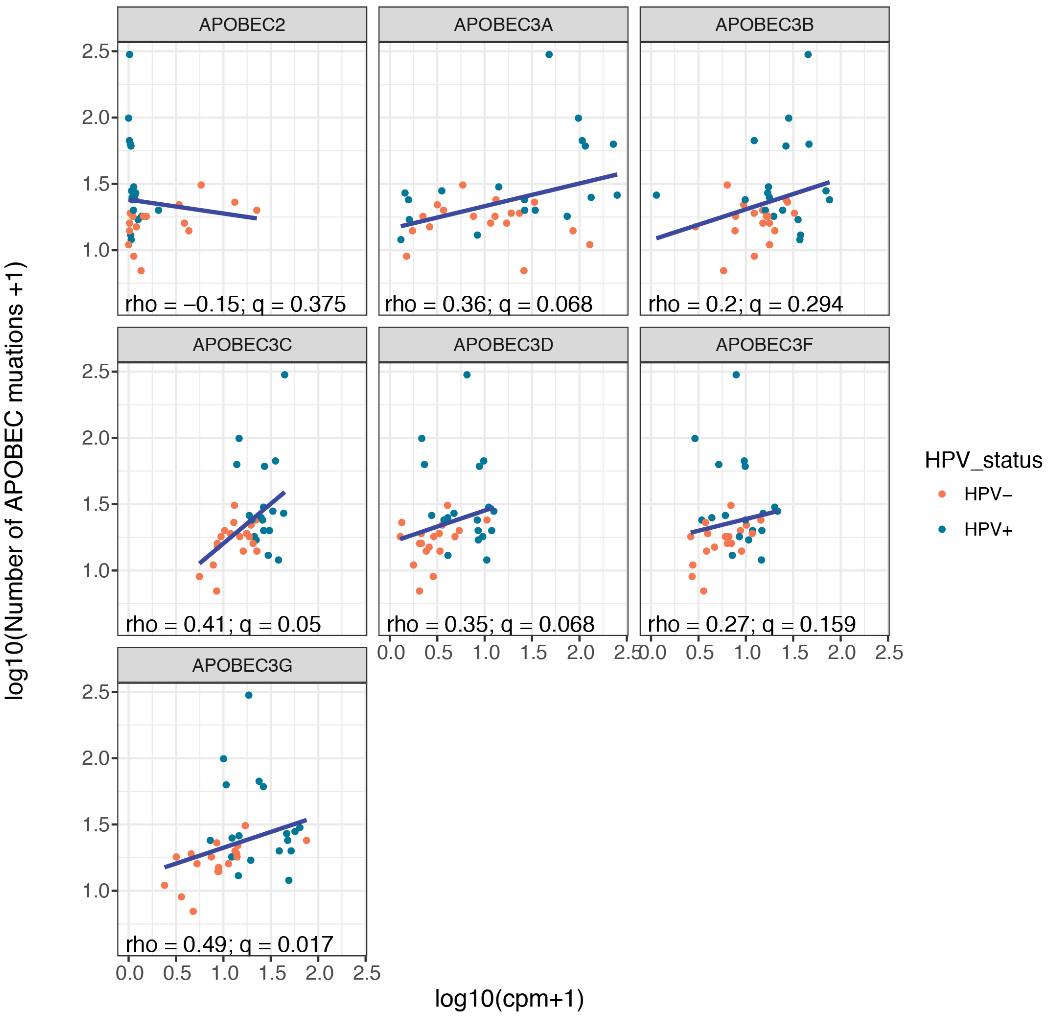
APOBEC2
APOBEC3A
APOBEC3B
APOBEC3C
APOBEC3D
APOBEC3F
APOBEC3G
rho = –0.15; q = 0.375
rho = 0.36; q = 0.068
rho = 0.2; q = 0.294
rho = 0.41; q = 0.05
rho = 0.35; q = 0.068
rho = 0.27; q = 0.159
rho = 0.49; q = 0.017
log10(Number of APOBEC muations +1)
log10(cpm+1)
HPV_status
HPV–
HPV+
